# Annotation-Efficient Deep Learning Model for Pancreatic Cancer Diagnosis and Classification Using CT Images: A Retrospective Diagnostic Study

**DOI:** 10.3390/cancers15133392

**Published:** 2023-06-28

**Authors:** Thanaporn Viriyasaranon, Jung Won Chun, Young Hwan Koh, Jae Hee Cho, Min Kyu Jung, Seong-Hun Kim, Hyo Jung Kim, Woo Jin Lee, Jang-Hwan Choi, Sang Myung Woo

**Affiliations:** 1 Graduate Program in System Health Science and Engineering, Division of Mechanical and Biomedical Engineering, Ewha Womans University, Seoul 03760, Republic of Korea; thanaporn.v@ewhain.net; 2 Center for Liver and Pancreatobiliary Cancer, National Cancer Center, Goyang 10408, Republic of Korea; deli4927@ncc.re.kr (J.W.C.); mrikyh@ncc.re.kr (Y.H.K.); lwj@ncc.re.kr (W.J.L.); 3 Department of Internal Medicine, Gangnam Severance Hospital, Yonsei University College of Medicine, Seoul 03722, Republic of Korea; jhcho9328@yuhs.ac; 4 Department of Internal Medicine, Kyungpook National University Hospital, Daegu 41944, Republic of Korea; minky1973@knu.ac.kr; 5 Department of Internal Medicine, Research Institute of Clinical Medicine of Jeonbuk National University—Biomedical Research Institute of Jeonbuk National University Hospital, Jeonju 54907, Republic of Korea; shkimgi@jbnu.ac.kr; 6 Department of Gastroenterology, Korea University Guro Hospital, Seoul 10408, Republic of Korea; hjkimmd@korea.ac.kr

**Keywords:** classification, deep learning, diagnosis, medical imaging, pancreatic cancer

## Abstract

**Simple Summary:**

In computer-assisted diagnostics for pancreatic cancer, attributes featuring irregular contours and indistinct boundaries on CT images present challenges in acquiring high-quality annotations. In response to this issue, we have devised an innovative self-supervised learning algorithm, engineered to enhance the differentiation of malignant and benign lesions. This innovation obviates the necessity for radiologist intervention, thus facilitating the precise classification of pancreatic cancer. By employing a pseudo-lesion segmentation self-supervised learning model, which capitalizes on automatically generated high-quality training data, we have managed to significantly elevate the performance of both convolutional neural network-based and transformer-based deep learning models.

**Abstract:**

The aim of this study was to develop a novel deep learning (DL) model without requiring large-annotated training datasets for detecting pancreatic cancer (PC) using computed tomography (CT) images. This retrospective diagnostic study was conducted using CT images collected from 2004 and 2019 from 4287 patients diagnosed with PC. We proposed a self-supervised learning algorithm (pseudo-lesion segmentation (PS)) for PC classification, which was trained with and without PS and validated on randomly divided training and validation sets. We further performed cross-racial external validation using open-access CT images from 361 patients. For internal validation, the accuracy and sensitivity for PC classification were 94.3% (92.8–95.4%) and 92.5% (90.0–94.4%), and 95.7% (94.5–96.7%) and 99.3 (98.4–99.7%) for the convolutional neural network (CNN) and transformer-based DL models (both with PS), respectively. Implementing PS on a small-sized training dataset (randomly sampled 10%) increased accuracy by 20.5% and sensitivity by 37.0%. For external validation, the accuracy and sensitivity were 82.5% (78.3–86.1%) and 81.7% (77.3–85.4%) and 87.8% (84.0–90.8%) and 86.5% (82.3–89.8%) for the CNN and transformer-based DL models (both with PS), respectively. PS self-supervised learning can increase DL-based PC classification performance, reliability, and robustness of the model for unseen, and even small, datasets. The proposed DL model is potentially useful for PC diagnosis.

## 1. Introduction

Pancreatic cancer (PC) is a highly fatal and malignant disease with a dismal prognosis. Despite recent advancements in surgical techniques, chemotherapy, and radiation therapy, the 5-year survival rate remains approximately 11% [1]. Notably, PC has become a common cause of cancer mortality. High mortality primarily results from advanced-stage cancer with metastatic disease at diagnosis [2]. The only hope of long-term survival in PC is if curative resection can be performed. However, PC is asymptomatic until the disease progresses to an advanced stage and, at diagnosis, only about 20% of cases are eligible for surgical resection [3,4,5,6].

Medical imaging plays several important roles in PC screening and early detection, preoperative evaluation and staging, differential diagnosis, follow-up, and treatment evaluation. A computed tomography (CT) scan is the most widely used imaging examination for the detection and staging of pancreatic carcinoma, given that its sensitivity ranges from 76–96%; notably, the sensitivity for larger tumors is higher than that for smaller tumors [7,8,9]. Generally, PC is characterized by abundant fibrous stroma and hypervascularity that account for the poor enhancement of the tumor compared with that of the surrounding pancreatic parenchyma on CT. These lead to poor diagnostic accuracy and sensitivity of tumor detection using CT. Moreover, at present, there is no standard imaging screening procedure, and the accuracy of PC detection and staging critically depends on the appropriate protocol, post-processing technique, and experience of the radiologist. In other words, detecting PC using only a CT scan is a very challenging task for radiologists, especially concerning small tumors in the early stages.

Artificial intelligence, particularly deep learning (DL), has demonstrated great promise for prognosis prediction in medical image analysis [10,11,12]. Major DL algorithms, such as the convolutional neural networks (CNNs) [13] and transformer architecture [14], have shown an impressive ability to extract complex visual information from medical images. However, despite the potential of DL, PC diagnosis using DL systems has not yet been actively investigated. Previous studies have demonstrated that DL could reduce the false diagnosis of PC on CT images as a second reader [15,16,17,18,19]. To achieve high-quality results and accurately generalize across multi-centers, CT equipment, and patient ethnicity, a large number of high-quality annotated training datasets are needed to allow deep networks to learn proper visual information for accurate classification. However, collecting a large volume of correctly annotated medical images for DL system development is a complex and expensive endeavor. Moreover, it is impractical to prepare such a well-curated pancreatic dataset, given that the accurate and early identification of PC on CT scans is still challenging, even for radiologists, because of the irregular contours and ill-defined margins of PC [20,21]. Therefore, it is difficult to achieve high prediction accuracy with a small training dataset for PC classification on CT scans, especially for small-sized tumors.

In this study, we proposed a novel self-supervised learning algorithm (pseudo-lesion segmentation [PS]) for PC classification using only CT scans. PS was designed to learn the prior visual representations of pancreatic CT scans in a supervised learning manner without requiring a radiologist or expert to annotate the ground truth label to achieve high performance on small training datasets, early stage cancer/small-sized tumors, and cross-ethnicity tests.

## 2. Related Work

DL techniques have exhibited promising results in the field of pancreatic cancer diagnosis using CT imaging [22,23,24]. A key contributor to the success of DL is the availability of extensive training data with manual labels supplied by radiologists. However, the scarcity of annotated medical images is a concern due to the requisite expertise of radiologists and the time-consuming nature of the task. In scenarios involving natural image classification, a prevalent approach is to leverage pre-existing visual representations learned from ImageNet, a large dataset of natural images used for classification tasks, employing pretrained weights [11,25]. Nonetheless, the use of ImageNet for learning visual representations in medical image classification is less than optimal because the visual representation learned from the natural image domain might not be suitable for the grayscale medical image domain. This unsuitability arises due to significant differences in feature distribution, spatial resolution, and output labels between the two domains.

Another notable technique for addressing the lack of labeled data is self-supervised learning. Self-supervised learning combines supervised and unsupervised learning approaches to learn semantically useful representations from pretext tasks. These tasks involve learning from unlabeled data by creating labels from the data for downstream tasks. Self-supervised learning enables the utilization of unlabeled domain-specific images by solving pretext tasks such as jigsaw puzzles, colorization tasks, and rotation prediction. This allows for the learning of more relevant feature representations for the image domain in downstream tasks like classification and segmentation [26,27,28]. In the field of medical image analysis, self-supervised learning with contrastive learning methods and image distortion pretext tasks has been employed to enhance the performance of various downstream tasks [29]. For example, Li et al. [30] successfully improved the performance of tumor classification by utilizing the feature representation learned through a pretext task of brain tumor segmentation. However, it is important to note that when tumors are not precisely segmented, the accuracy of tumor classification using the learned features is not guaranteed [30]. Moreover, considering the significant variation in annotation tasks between raters [31], which can lead to different conclusions regarding medical diagnoses [32], it becomes challenging to ensure high-quality annotations.

In this study, we created a pseudo-lesion using an undefined atypical shape that mimicked the shape of a tumor. Because the atypical shape is composed of a random combination of a plurality of simple shapes, it can be easily generated and a variety of complex and differing types of lesions, such as actual tumors, can be formed. Previously, a pseudo-lesion was created in the form of a simple geometric shape, but this simple shape was very weak in its ability to simulate the complex shape of a real tumor [18]. However, in model observer studies for image quality evaluation, a more realistic tumor or lesion was synthesized and inserted into the CT image. Such realistic tumor shapes were created by synthesizing the actual lesion shapes and organ-specific background textures present in organs such as the breast, liver, or lungs [33,34]. If our proposed self-supervised algorithm learns feature representation using more realistic tumor shapes, tumor classification performance may be improved. However, it is expected that the complex shape of different tumors for each organ or lesion reflecting the noise characteristics different for each system has poor reproducibility, making it difficult to apply to new organs or systems that are not well known.

## 3. Materials and Methods

### 3.1. Ethics

All procedures were performed in compliance with the relevant laws and institutional guidelines. The study was approved by the Institutional Review Board of the National Cancer Center (NCC) (2020-0327). The requirement for written informed consent was waived owing to the retrospective nature of the study.

### 3.2. Patient and Data Collection

In this retrospective diagnostic study, we analyzed CT images acquired from the following datasets: the National Information Society Agency (NIA)-funded Medical Big Data Construction Project, the Medical Segmentation Decathlon, and the Cancer Imaging Archive (TCIA). The NIA-funded project included CT images of PC and normal pancreatic tissues from the NCC and seven general tertiary hospitals in South Korea. PC was defined as histologically or cytologically confirmed pancreatic adenocarcinoma. Benign pancreatic disease included pancreatic cystic lesions and acute or chronic pancreatitis, with a 1-year follow-up period. Images in which no lesions were observed were selected based on the radiologist’s report (a negative or unremarkable pancreas) as the normal pancreas set from participants who underwent a health checkup or treatment for anything other than pancreatic disease.

In the NIA-funded project, CT images were obtained in the portal venous phase (70 s after intravenous contrast injection) or pancreatic phase (40 s after contrast injection). In the labelling of the lesions, blood vessels were excluded as much as possible. In cases of pancreatitis, the entire lesion, including the peripancreatic infiltration was labelled. The Medical Segmentation Decathlon and TCIA dataset consists of portal venous phase CT images with the resolution of 512 × 512 pixels with varying pixel sizes and slice thickness between 1.5–2.5 mm, acquired on Philips and Siemens MDCT scanners (120 kVp tube voltage).

**Figure 1:**
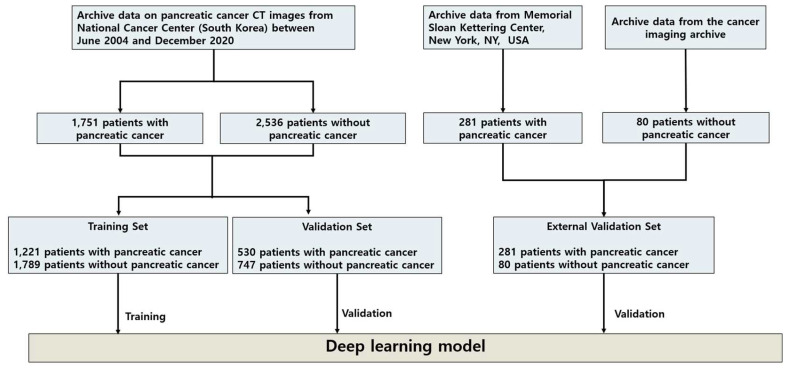
Flowchart of dataset collection for developing the proposed PS self-supervised learning method. CT image datasets from the NCC, Korea, which were collected from seven general tertiary hospitals in South Korea, were randomly divided into 3010 patients for the DL model training set and 1277 patients for the internal validation set. For the external validation set, two pancreatic CT image datasets from the Memorial Sloan Kettering Center, New York, NY, USA, and TCIA were combined for the DL model’s evaluation. Abbreviations: CT, computed tomography; DL, deep learning; NCC, National Cancer Center; PS, pseudo-lesion segmentation; TCIA, the Cancer Imaging Archive.

A flowchart describing the research process is presented in Figure 1.

For algorithm development, we used data collected from the NCC for the training set and validation set and those from the Medical Segmentation Decathlon and TCIA as the cross-ethnicity external validation set. For the training set and validation set, we utilized the CT images of 4287 patients that were collected between June 2004 and December 2020. All CT scans were carefully reviewed by two experienced radiologists with >5 years of experience in pancreatic imaging. A total of 3010 patients comprised the training set, and 1277 patients comprised the validation set. The CT images of 361 patients from two external sources comprised the cross-ethnicity external validation set. Detailed baseline characteristics are presented in Table S1.

### 3.3. Development of PS Self-Supervised Learning

**Figure 2:**
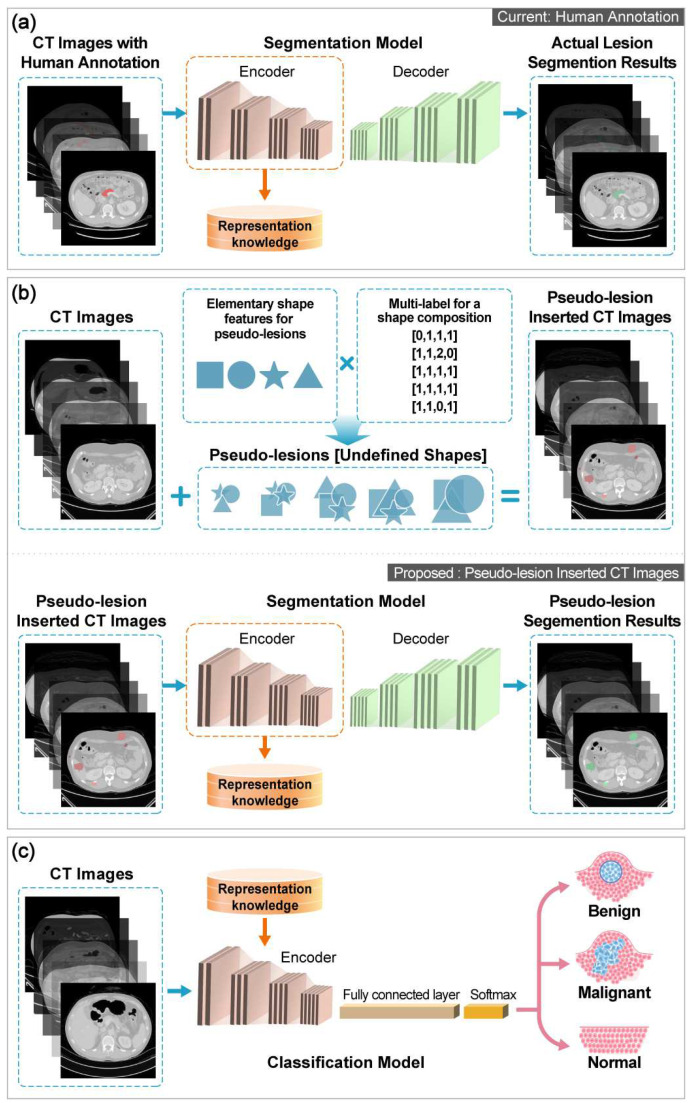
Overview of self-supervised learning in this study. (**a**) Actual lesion segmentation pretext task in which the lesion ground truth label is indicated by an experienced radiologist. (**b**) Proposed PS pretext task. (**c**) Fine-tuning PC classification DL model. Abbreviations: CT, computed tomography; DL, deep learning; PC, pancreatic cancer; PS, pseudo-lesion segmentation.

The self-supervised learning algorithm is a technique for solving limited annotated data scenarios in both the natural and medical imaging domains. We developed a novel self-supervised learning algorithm, PS, to overcome a small-size training dataset problem and improve DL system performance in early stage PC and cross-ethnicity tests. The proposed PS was designed to learn prior to the semantic representation (i.e., pancreas-related visual representation) from the PC classification data itself via the PS task. Unlike previous research [30], which learned the prior knowledge from the dataset in which the annotated lesion regions were defined by radiologists (Figure 2a), our PS learned the representation from the automatically generated annotation dataset without requiring humans to annotate using labels (Figure 2b).

Notably, the classification accuracy of previous research depends on the correctness of the tumor region annotation defined by humans [30]; therefore, it cannot guarantee classification performance when the tumor region is not precisely segmented. Moreover, considering that annotation tasks are often prone to significant variation between raters [31] and that the variation results in different conclusions regarding medical diagnosis [32], it is difficult to secure high-quality annotations. Nonetheless, with an automatically generated annotated dataset, the correctness of the annotation of our proposed PS can be guaranteed.

The PS consisted of three steps. We first automatically generated an annotation called pseudo-lesion for prior representation learning via a segmentation task (pretext task) and inserted it into the pancreatic CT scans. The details of the pseudo-lesion generation and insertion are described in Methods S1. An example of the pseudo-lesion inserted CT images is shown in Figure S1. We created a pseudo-lesion using an undefined atypical shape that mimicked the shape of a tumor. Because the atypical shape is composed of a random combination of a plurality of simple shapes, it can be easily generated, and complex and varying types of lesions, such as actual tumors, can be formed. Subsequently, a DL network was trained to learn the pancreas and tumor-related visual representation by segmenting the pseudo-lesion regions in the generated dataset. Finally, we fine-tuned the pretrained DL network for PC classification in a supervised learning manner (Figure 2c).

### 3.4. Training of DL Models

We incorporated the proposed PS with state-of-the-art DL models, including a CNN-based model named ShuffleNet V2 [13] and a transformer-based model named Pyramid Vision Transformer (PVT) [14] for PC classification using CT scans. Note that, as demonstrated in Table 1, ShuffleNet V2 and PVT showed the best PC classification performance among the latest CNN-based models and transformer-based models tested, respectively, so they were selected as the baseline models. All DL models were trained using CT images collected from 3010 patients and validated on two datasets: an internal validation set (1277 patients) and an external validation set (361 patients). Moreover, we compared the performance of the DL model with and without PS to evaluate the performance of the proposed PS. An implementation detail of the DL models in the experiments is described in the Supplementary Materials (Methods S2 and Table S2). Furthermore, to explore the robustness of the self-supervised learning with PS for small datasets, we performed an experiment involving various training image dataset sizes of 10%, 25%, 50%, 75%, and 100% of the entire training dataset.

### 3.5. Statistical Analysis

The predictive labels with reference to the ground truth labels were depicted as confusion matrices, which were used to calculate the accuracy, sensitivity, specificity, precision, F1 score, and area under the receiver operating characteristic curve (AUC). Furthermore, the Clopper–Pearson method was used to calculate the 95% confidence interval (CI) for accuracy, sensitivity, specificity, and precision. All computations and statistical analyses were performed using the scikit-learn package, version 0.34, in Python, version 3.7 (Python Software Foundation). These tasks were carried out in an environment equipped with an NVIDIA Titan Xp GPU (NVIDIA Corp., Silicon Valley, USA).

## 4. Results

### 4.1. Clinicopathological Data

A total of 4287 patients underwent CT imaging between June 2004 and December 2020 in Korea (mean (standard deviation (SD)) age: 58.9 (13.5) years), and CT images from an external source of 361 patients were utilized in this study. Data from 3010 patients (mean (SD) age: 58.9 (13.5) years) were labeled by experienced radiologists and used for training the DL model. The two validation sets encompassed the CT images of 1277 patients (mean (SD) age: 58.9 (13.4) years) from the NCC and 361 patients from two open-source datasets from research institutions in the United States (Figure 1), namely the Medical Segmentation Decathlon dataset from the Memorial Sloan Kettering Cancer Center (281 patients with PC) (preprint) [40] and the TCIA dataset from the United States National Institutes of Health Clinical Center (80 patients with normal pancreas) [41].

### 4.2. PC Classification

#### 4.2.1. State-of-the-Art DL Models for PC Classification

In order to select the state-of-the-art DL model to incorporate with our proposed PS, we performed an experiment to evaluate the performance of each DL classification model, including CNN and transformer-based architecture, on the validation set of pancreatic cancer dataset. Table 1 demonstrates that ShuffleNet V2 and PVT achieved the highest accuracy for CNN and transformer-based architecture, respectively, with accuracies of 93.6% (92.1–94.8%) and 90.6% (88.8–92.1%).

#### 4.2.2. Impact of the PS on PC Classification on the Internal Validation Dataset

We conducted the experiments to evaluate the performance of the proposed PS incorporated with CNN-based and transformer-based DL architecture, i.e., ShuffleNet V2 and PVT, respectively, by comparing DL models with and without PS. As shown in Table 2, the CNN-based DL model with PS achieved a PC classification accuracy of 94.3% (95% CI: 92.8–95.4%), which was 0.7% higher than the accuracy of 93.6% (95% CI: 92.1–94.8%) achieved by the CNN-based model without PS.

**Figure 3:**
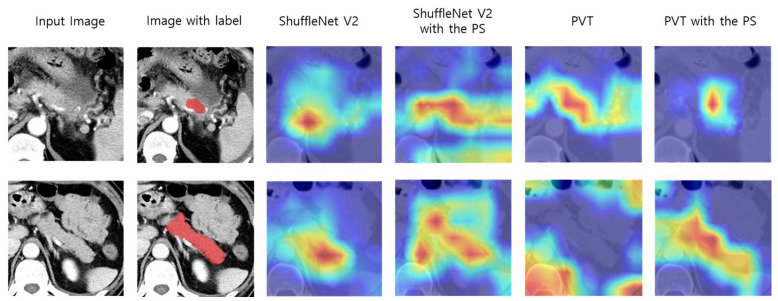
Comparison of representative CT images with heat map overlay for tumor regions and pancreatic regions of DL models with and without PS. Abbreviations: CT, computed tomography; DL, deep learning; PS, pseudo-lesion segmentation; PVT, pyramid vision transformer.

The CNN-based model with PS demonstrated improved sensitivity, specificity, precision, F1 score, and AUC compared to the model without PS. Additionally, the transformer-based model with PS exhibited even greater enhancements in performance, surpassing the transformer-based model without PS by 5.1% in accuracy, 1.9% in sensitivity, 3.2% in specificity, 15.4% in precision, 0.15 in F1 score, and 0.07 in AUC. From these results, implementing the proposed PS can improve all evaluation metrics on both CNN-based and transformer-based DL models for PC classification. In other words, PS can improve the prediction reliability of the DL models to be more similar to experienced radiologists (ground truth). Furthermore, Figure 3 shows representative CT images overlaid with heat maps produced by the gradient-weight class activation map (Grad-CAM) [42].

The red and yellow regions on the heat maps represent areas activated by the DL models and have the greatest predictive significance. The results show that incorporating PS with the DL models increases the model’s ability to capture the tumor pixel-wise region for CT images with PC and the pancreatic pixel-wise region for CT images for a normal class.

### 4.3. External Validation Set Classification

A practical DL model should generalize well to the unseen datasets of different ethnic groups obtained from different institutions. We explored the robustness of the DL models to the unseen image source by evaluating the DL models that were trained on the internal training set and validated on the external validation set. The external validation set contained CT images from two different sources and different characteristics (i.e., a patient’s ethnicity) from the internal dataset. The experiment results on the external validation set are summarized in Table 3. The CNN-based model with PS achieved higher accuracy (82.5% [95% CI: 78.3–86.1%]), sensitivity (81.7% [95% CI: 77.3–85.4%]), specificity (100.0% [95% CI: 81.7–100.0%]), precision (100.0% [95% CI: 98.6–100.0%]), F1 score (0.90), and AUC (0.61), compared to the CNN-based model without PS (80.9% [95% CI: 76.5–84.6%], 80.3% [95% CI: 75.8–84.1%], 100.0% [95% CI: 74.1–100.0%], 100.0% [95% CI: 98.6–100.0%], 0.89, and 0.57, respectively). In addition, the transformer-based model with PS increased the accuracy by 4.7%, sensitivity by 4.2%, specificity by 4.8%, precision by 0.4%, F1 score by 0.03, and AUC by 0.18, with an accuracy of 87.8% (95% CI: 84.0–90.8%), sensitivity of 86.5% (95% CI: 82.3–89.8%), specificity of 100.0% (95% CI: 90.4–100.0%), F1 score of 0.93, and AUC of 0.80, from the baseline model without PS. These results implied that PS self-supervised learning can enhance the robustness of the DL models to unseen datasets.

### 4.4. Early Stage PC Detection

Table 4 presents the DL models’ early stage PC detection performance, which is challenging to visualize in CT images, even for the radiologist, and the accurate diagnosis of early stage PC can increase the survival rate of the patients.

The CNN-based model with PS outperformed the model without PS with an accuracy of 54.0% (95% CI: 44.8–57.8%) for PC stage T1 and 76.9% (95% CI: 74.6–79.0%) for PC stage T2. Furthermore, the transformer-based model with PS achieved an accuracy of 55.3% (95% CI: 48.8–61.8%) for PC stage T1 and 75.2% (95% CI: 72.7–77.6%) for PC stage T2, which were higher than those of the model without PS that achieved an accuracy of 50.4% (95% CI: 47.0–56.9%) for PC stage T1 and 67.1% (95% CI: 64.6–69.9%) for PC stage T2. As shown in Figure S2, the DL models with PS are more accurately focused on predicting tumor regions than the models without PS. In other words, the incorporated PS with DL models can increase the prediction accuracy and ability of the model to focus on the tumor regions more accurately compared to the model without PS.

### 4.5. Performance Changes Depending on the Size of the Annotated Dataset

**Figure 4:**
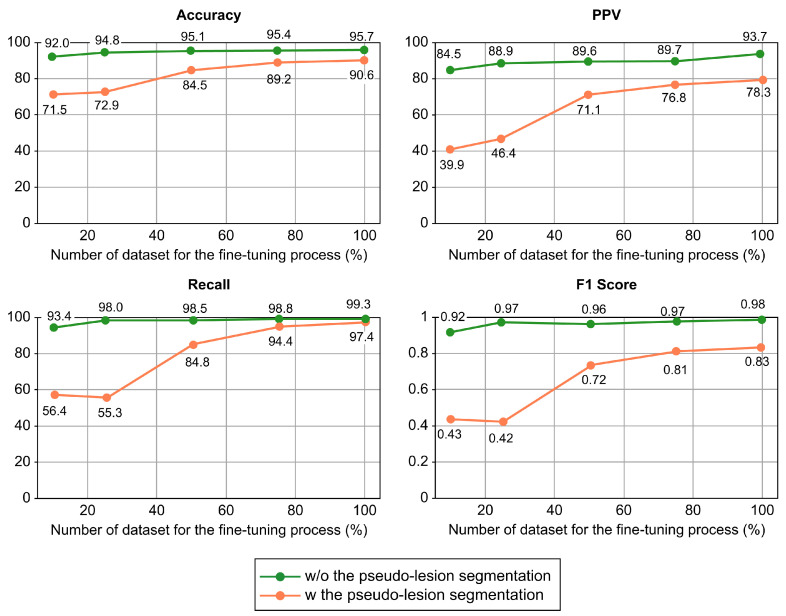
Performance comparison of a transformer-based DL model with and without our proposed self-supervised learning method on a varying number of datasets of fine-tuning process. Abbreviations: DL, deep learning; PPV, pyramid vision transformer.

To evaluate the robustness of the self-supervised learning with PS for small datasets, we randomly sampled 10%, 25%, 50%, and 75% of the entire training dataset and trained PVT with and without PS using these selected datasets. Figure 4 presents performance changes depending on the size of the annotated dataset. PS shows a remarkable increase in the classification performance of the DL model for small datasets (10% and 25% datasets).

Specifically, by adopting the PS, when the DL model was trained with only 10% of the dataset, the prediction accuracy and sensitivity improved by 20.5% and 37.0%, respectively. This suggests that the implementation of PS could help overcome the problem of low DL model accuracy in situations with limited dataset availability.

## 5. Discussion

Several challenges in PC diagnosis exist. For instance, most PCs have poorly enhanced, ill-defined masses with indistinct borders from the surrounding tissues on CT [20]. Occasionally, there are no apparent lesions, and only pancreatic duct dilatation, distal pancreatic atrophy, abnormal pancreatic contour, and ductal interruption can be observed [43]. Therefore, radiologists’ expertise and experience in centers dealing with large numbers of PC cases affect the accuracy of their interpretations [44]. As such, it is challenging to accurately segment PC in CT images, making it extremely difficult to build a large amount of high-quality annotated datasets. This acts as a major barrier to developing a DL-based model for PC diagnosis.

In this study, we successfully developed a novel self-supervised learning algorithm (PS), which enhances the PC classification performance of the DL models and generalizes well on new image sources of different patient ethnicities acquired from multiple centers. The tumor location predicted by the DL models with our PS algorithm showed better correspondence with radiologist labeling than the DL models without PS. This supports the potential usefulness of the DL model with PS, particularly in pre-referral centers or by less experienced radiologists involved in PC diagnosis. Furthermore, the proposed PS demonstrated promising classification performance, even with small, annotated training datasets. Compared to the performance of DL models alone, the DL models with the proposed PS trained with 10% of the dataset showed 20.5% and 37.0% enhanced accuracy and sensitivity, respectively, which means that we are also able to build a successful model with small datasets with this technique.

In addition, the DL models with PS self-supervised learning demonstrated the feasibility of using the DL models with PS to detect early stage PC by outperforming the DL models without PS. Generally, patients with an early T1/T2 stage have a poor prognosis compared to those with a late stage [45]. Therefore, the prompt detection of early stage PC is imperative for early interventions and improved prognosis. However, tumors <2 cm are often unremarkable on CT and approximately 40% are undetected at diagnosis [46], with a reported sensitivity as low as 58–77% [47]. We found that the DL model with the proposed PS achieved comparable results to that of previously reported interpretations by experienced radiologists and was superior to other learning models in both T1 and T2 tumors, suggesting that it can reduce overlooked or missed diagnoses of early stage PC, potentially resulting in improved patient outcomes.

This work demonstrates the improved robustness of DL models for new image sources of different ethnicities obtained from multi-centers. Compared to the performance of DL models without PS, the DL models with PS achieved higher performance on external validation, which is the combination of CT images from two different open-source datasets from the United States. The lower accuracy and sensitivity of the DL models with PS in the external validation set compared with the internal validation set may contribute to the differences in race/ethnicity and diverse scanners and settings. The participants in our internal dataset were entirely Asian patients, and the external dataset consisted of two different open-source datasets from the United States. The pancreatic content is one of the major factors influencing race/ethnicity differences [16,48,49]. Furthermore, diverse scanners at different institutions may also decrease sensitivity and accuracy. However, since the pancreatic CT protocol is usually recommended for diagnosing pancreatic disease, this difference between centers could be minimized. Rather, CT images with multicenter technical variations from a large number of patients reflect a real clinical practice situation well, suggesting the potential generalizability of our PS model to real-world clinical practice. Additionally, the external performance showed only a modest decrease compared with previous DL algorithms, despite ethnic and technological differences [50].

Our study has some limitations. First, without further cancer prediction results from the variable experience levels of radiologists, the model’s ability to reduce the number of overlooked lesions could not be substantiated. Second, the training dataset was collected from seven general tertiary hospitals in only one country (Republic of Korea); thus, there was little ethnic variation. Therefore, the proposed DL method that was tested on the external validation set, acquired from a different distribution in terms of the instrument setting and patient characteristics, such as age and ethnicity, could not achieve as high accuracy as when testing with an internal validation set. Thus, to increase the practical feasibility of the proposed method, we plan to develop a method that increases the robustness of the model for testing with an external dataset.

## 6. Conclusions

In conclusion, we developed a DL-based automatic classification algorithm that increases the performance of state-of-the-art DL algorithms and outperforms other DL algorithms in multiclass, binary, and early stage cancer classifications. Moreover, we demonstrated that our proposed method could potentially increase the robustness of the model when trained with a small dataset. Furthermore, the proposed PS self-supervised learning enhances the ability of the model to classify PC from outside image sources or different scanners.

## Figures and Tables

**Table 1:** Performance comparison of the state-of-the-art deep learning models using the internal validation set.

DL Model	Accuracy (95% CI)	Sensitivity (95% CI)	Specificity (95% CI)	Precision (95% CI)	F1 Score
CNN-based architecture					
ResNet 101 [35]	90.2% (89.9–90.6%)	78.6% (77.6–79.5%)	91.1% (90.7–91.6%)	80.4% (79.5–81.4%)	0.79
ResNeXt-101 [36]	83.5% (83.0–83.9%)	62.7% (61.7–63.7%)	84.9% (84.3–85.4%)	64.3% (63.0–65.5%)	0.62
ResNeSt [37]	84.3% (83.9–84.7%)	63.2% (62.2–64.1%)	84.5% (84.0–85.0%)	67.9% (66.7–69.2%)	0.64
ShuffleNet V2	93.6% (92.1–94.8%)	90.6% (87.9–92.9%)	95.5% (93.8–96.8%)	93.9% (91.5–95.6%)	0.92
Transformer-based architecture					
MiT [38]	85.4% (85.0–85.8%)	64.5% (63.5–65.5%)	84.7% (84.2–85.2%)	71.8% (70.5–73.0%)	0.66
PiT [39]	82.8% (82.3–83.2%)	56.3% (55.5–57.2%)	80.2% (79.7–80.7%)	68.8% (67.2–70.4%)	0.56
PVT	90.6% (88.8–92.1%)	97.4% (95.3–98.6%)	87.5% (85.1–89.6%)	78.3% (74.4–81.7%)	0.83

Abbreviations: CI, confidence interval; CNN, convolutional neural network; DL, deep learning; PVT, pyramid vision transformer; PiT, pooling-based vision transformer; MiT, mix transformer.

**Table 2:** Performance comparison of the DL models with and without PS self-supervised learning for the internal validation set.

DL Model	Accuracy (95% CI)	Sensitivity (95% CI)	Specificity (95% CI)	Precision (95% CI)	F1 Score	AUC
CNN-based architecture						
ShuffleNet V2	93.6% (92.1–94.8%)	90.6% (87.9–92.9%)	95.5% (93.8–96.8%)	93.9% (91.5–95.6%)	0.92	0.93
ShuffleNet V2 + PS	94.3% (92.8–95.4%)	92.5% (90.0–94.4%)	95.8% (94.2–97.1%)	94.4% (92.2–97.1%)	0.93	0.94
Transformer-based architecture						
PVT	90.6% (88.8–92.1%)	97.4% (95.3–98.6%)	87.5% (85.1–89.6%)	78.3% (74.4–81.7%)	0.83	0.88
PVT + PS	95.7% (94.5–96.7%)	99.3% (98.4–99.7%)	90.7% (88.0–92.9%)	93.7% (91.8–95.2%)	0.98	0.95

Abbreviations: AUC, area under the receiver operating characteristic curve; CI, confidence interval; CNN, convolutional neural network; DL, deep learning; PS, pseudo-lesion segmentation; PVT, pyramid vision transformer.

**Table 3:** Performance comparison of the DL models with and without PS self-supervised learning for external validation set.

DL Model	Accuracy (95% CI)	Sensitivity (95% CI)	Specificity (95% CI)	Precision (95% CI)	F1 Score	AUC
CNN-based architecture						
ShuffleNet V2	80.9% (76.5–84.6%)	80.3% (75.8–84.1%)	100.0% (74.1–100.0%)	100.0% (98.6–100.0%)	0.89	0.57
ShuffleNet V2 + PS	82.5% (78.3–86.1%)	81.7% (77.3–85.4%)	100.0% (81.7–100.0%)	100.0% (98.6–100.0%)	0.90	0.61
Transformer-based architecture						
PVT	83.1% (78.9–86.6%)	82.3% (77.9–86.6%)	95.2% (77.3–99.1%)	99.6% (98.0–99.9%)	0.90	0.62
PVT + PS	87.8% (84.0–90.8%)	86.5% (82.3–89.8%)	100.0% (90.4–100.0%)	100.0% (98.6–100.0%)	0.93	0.80

Abbreviations: AUC, area under the receiver operating characteristic curve; CI, confidence interval; CNN, convolutional neural network; DL, deep learning; PS, pseudo-lesion segmentation; PVT, pyramid vision transformer.

**Table 4:** Accuracy of the DL models with and without PS self-supervised learning for early stage PC.

DL Model	Accuracy (95% CI)		
	Stage T1	Stage T2	All Stages
CNN-based architecture			
ShuffleNet	51.3% (44.8–57.8%)	68.4% (65.9–70.8%)	93.6% (92.1–94.8%)
ShuffleNet + PS	54.0% (47.5–60.4%)	76.9% (74.6–79.0%)	94.3% (92.8–95.4%)
Transformer-based architecture			
PVT	50.4% (47.0–56.9%)	67.1% (64.6–69.9%)	90.6% (88.8–92.1%)
PVT + PS	55.3% (48.8–61.8%)	75.2% (72.7–77.6%)	95.7% (94.5–96.7%)

Abbreviations: CI, confidence interval; CNN, convolutional neural network; DL, deep learning; PC, pancreatic cancer; PS, pseudo-lesion segmentation; PVT, pyramid vision transformer.

## Data Availability

The datasets generated during and/or analyzed during the current study are available at the NIA-funded Medical Big Data Construction Project (https://aihub.or.kr/, accessed on 25 May 2023), the Medical Segmentation Decathlon (http://medicaldecathlon.com/), and TCIA (https://cancerimagingarchive.net/). All computer codes used for modeling and data analysis were stored at https://github.com/Thanaporn09/Cancer_self_transformer.git. The data generated in this study can be made accessible upon a reasonable request directed to the corresponding authors from each respective dataset repository.

## Supplementary Materials

The following supporting information can be downloaded at: https://www.mdpi.com/article/10.3390/cancers15133392/s1, Method S1: Current solutions in limited annotated data scenarios (including self-supervised learning); Method S2: Pretext task training dataset generation; Method S3: Deep learning model implementation/training/validation; Table S1: Demographic and clinical characteristics; Table S2: The network configuration for the DL model; Figure S1: Example of the segmentation pretext task input images (pancreatic cancer computed tomography [CT] scans); Figure S2: Comparison of representative computed tomography images with heat map overlay for tumor regions and pancreatic regions of deep learning models with and without pseudo-lesion segmentation of early stage pancreatic cancer. Reference [51] is found in Supplementary Materials.
