# Supplementary material for: Annotation-Efficient Deep Learning Model for Pancreatic Cancer Diagnosis and Classification Using CT Images: A Retrospective Diagnostic Study
Source: Cancers (Basel). 2023 Jun 28;15(13):3392. doi: 10.3390/cancers15133392 (PMC10340780; doi:10.3390/cancers15133392)
Supplement: Supplementary file 1 [file cancers-15-03392-s001.zip › cancers-2445000-supplementary.pdf]

Supplementary Materials:

Annotation-Efficient Deep Learning Model for Pancreatic Cancer Diagnosis and Classification Using CT Images: A Retrospective Diagnostic Study

Thanaporn Viriyasaranon [1,†], Jung Won Chun [2,†], Young Hwan Koh [2], Jae Hee Cho [3], Min Kyu Jung [4], Seong-Hun Kim [5], Hyo Jung Kim [6], Woo Jin Lee [2], Jang-Hwan Choi [1,*] and Sang Myung Woo [2,*]

Methods S1: Pretext task training dataset generation

To generate the pseudo-lesion (undefined shape), we randomly selected the elementary shape features (i.e., circle, triangle, square, and star) and combined the selected shape with the random number of each elementary shape, size, and position. We then generated images for the pseudo-lesion segmentation task dataset by inserting the pseudo-lesion into the human tissue region of the computed tomography (CT) images from the pancreatic cancer classification task. To overcome the difficulty of detecting early-stage cancer, we set the size of the pseudo-lesion to ≤2 cm (75 pixels), which is the tumor size of pancreatic cancer stage T1 [51]. Therefore, we generated the pseudo-lesion with four shape diameter sizes (i.e., 10, 20, 30, and 60 pixels) for the pretext task dataset. Moreover, we applied various brightness and Gaussian filters with a kernel size of 4.0 and a sigma value of 1.0 to blur the edges and textures of the inserted shape and blend it with the human tissue regions in the CT images. For each CT image, the brightness and position of an undefined shape were randomly selected and inserted.

Methods S2: Deep learning model implementation/training/validation

We implemented our deep learning system using both convolutional neural network and transformer architecture with a PyTorch-based open-source image classification toolbox (MMClassification version 0.19.0). The model was trained to map 2D slices of CT images without any exclusion to assess tumor classification. Furthermore, we cropped the tumor region of each slice from 512 × 512 to 224 × 224 pixel-size images for all experiments. Moreover, we augmented our training data with random flips of the original images and optimized the training model using an AdamW optimizer with a learning rate of 8e-5 for 50 epochs.

Table S1. Demographic and clinical characteristics.

	Training set	Validation set	External validation set
Patients	3,010	1,277	361
Age (years)	58.9±13.5 (17–96)	58.9±13.4 (20–94)	–
<40	272 (9.0%)	95 (7.4%)	–
40–49	452 (15.0%)	217 (17.0%)	–
50–59	751 (24.9%)	335 (26.2%)	–
60–69	807 (26.8%)	328 (25.7%)	–
≥70	728 (24.2%)	302 (23.6%)	–
Race			
Korean	2,983 (99.1%)	1,259 (98.6%)	0 (0.0%)
Non-Korean	27 (0.9%)	18 (1.4%)	361 (100.0%)
Sex			
Male	1,374 (45.6%)	582 (45.6%)	–
Female	1,636 (54.3%)	695 (54.4%)	–
Tumor type			
Cancer	1,221 (40.6%)	530 (41.5%)	281 (77.8%)
Stage T1	132 (10.8%)	63 (11.9%)	–
Stage T2	456 (37.3%)	197 (37.2%)	–
Stage T3	288 (23.6%)	124 (23.4%)	–
Stage T4	345 (28.2%)	146 (27.5%)	–
Normal	1,789 (59.4%)	747 (58.5%)	80 (22.2%)

Table S2. The network configuration for the DL model.

DL Model	Optimizer	Learning rate	Weight Decay	#epochs
ShuffleNet V2	SGD	0.003	0.0001	50
ShuffleNet V2 + PS	SGD	0.003	0.0001	50
PVT	AdamW	8e-5	0.01	50
PVT+PS	AdamW	8e-5	0.01	50

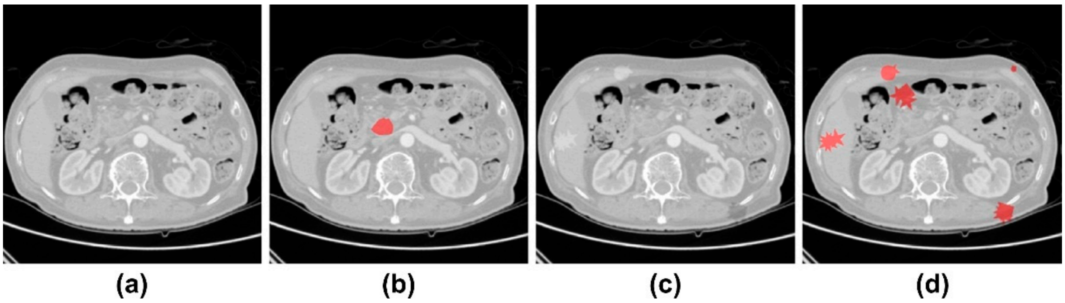

Figure S1. Example of the segmentation pretext task input images (pancreatic cancer computed tomography [CT] scans). (a) Original CT image. (b) CT image with a marked lesion. (c) CT image with pseudo-lesions. (d) CT image with marked pseudo-lesions.

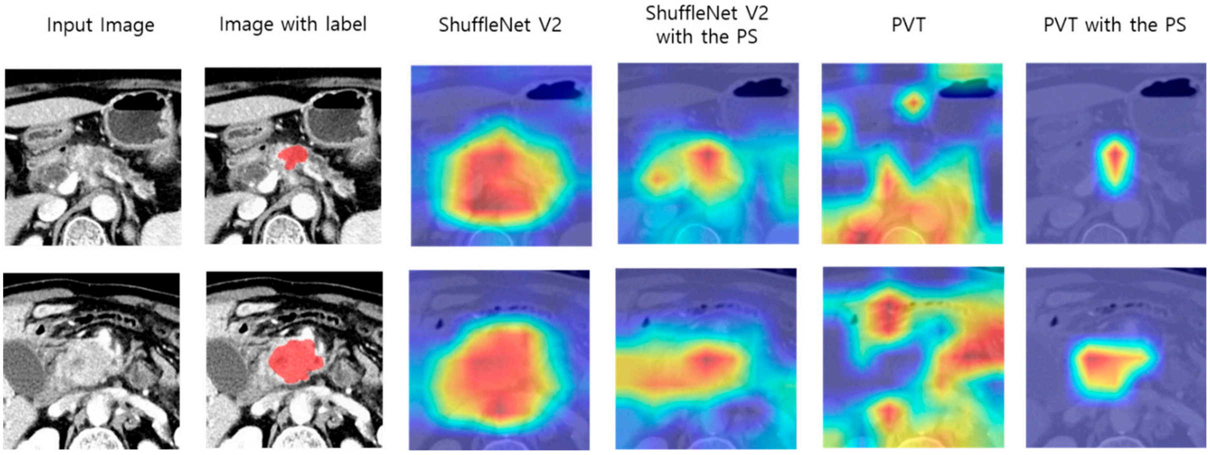

Figure S2. Comparison of representative computed tomography images with heat map overlay for tumor regions and pancreatic regions of deep learning models with and without pseudo-lesion segmentation of early-stage pancreatic cancer. Abbreviations: PS, pseudo-lesion segmentation; PVT, Pyramid Vision Transformer.
